# Closing the Gaps in Understanding PFAS Toxicology and Metabolism

**DOI:** 10.3390/toxics13010019

**Published:** 2024-12-27

**Authors:** Denise K. MacMillan, Barbara A. Wetmore, Subham Dasgupta, William S. Baldwin

**Affiliations:** 1 Center for Computational Toxicology and Exposure, Office of Research and Development, U.S. Environmental Protection Agency (EPA), Durham, NC 27709, USA; wetmore.barbara@epa.gov; 2 Department of Biological Sciences, Clemson University, Clemson, SC 29634, USA; subhamd@clemson.edu (S.D.); baldwin@clemson.edu (W.S.B.)

## 1. Introduction

Per- and polyfluoroalkyl substances (PFAS) are nearly ubiquitous and found in rivers, soils, atmosphere, food packaging, clothing, cosmetics, commercial products, homes, drinking water, and humans and other organisms [1]. The high number, variety of chemical properties, and widespread occurrence of PFAS complicates research into additional understanding of environmental and human health impacts. There is strong support from the database of published literature regarding the human health effects after exposure to the well-studied PFAS, perfluorooctanoic acid (PFOA) or perfluorooctanesulfonic acid (PFOS), summarized in the recently finalized United States Environmental Protections Agency (EPA) toxicity assessments [2,3]. Based on the health effects associated with human health, phase outs [4,5,6] and risk reduction [7] have resulted. However, gaps in toxicity data for legacy and emerging PFAS in this structurally diverse class persist. Gaining a better understanding of the potentially toxic effects of PFAS mixtures is also a critical data gap because of co-exposure to multiple diverse PFAS [8]. The Special Issue of *Toxics* entitled “PFAS Toxicology and Metabolism” expands our knowledge of the environmental and human health effects of PFAS by providing original research and reviews that describe new methods, toxicokinetics, fate and bioaccumulation, metabolism, transport, toxicity, comparative toxicity, and adverse effects of individual PFAS and PFAS mixtures.

## 2. Discussion

Fate and bioaccumulation are cornerstone issues related to PFAS toxicity because many PFAS have long half-lives. Dawson et al. [9] provides new information including a machine-learning model for estimating the half-lives of multiple PFAS across several species such as human, monkey, rat, and mouse. The model separates PFAS with half-lives over 2 months from those eliminated in hours, days, and weeks. This is crucial information due to the infeasibility of testing every PFAS in every human- or environmentally-relevant model. Further research that tests and compares different PFAS with diverse half-lives may refine the model further [9]. Kolanczyk et al. [10] investigated PFAS biotransformation across multiple species, including poultry, mammals, earthworms, fungus, and bacteria, and found that biotransformation pathways are relatively well conserved. Given that there are few PFAS biotransformation studies specifically in fish, comparative studies may help inform metabolite prediction across other species [10].

Several other studies also evaluated PFAS bioaccumulation or toxicokinetics [11,12,13,14,15] by assessing in vitro hepatic clearance. Crizer et al. [15] investigated toxicokinetics for 54 legacy and emerging PFAS. While 35 PFAS exhibited little clearance, high clearance rates were observed for fluorotelomers and perfluorosulfonamides, both of which are known to biotransform to more stable compounds with lower clearance. Kreutz et al. [12] evaluated the toxicokinetics (i.e., plasma protein-binding and hepatic clearance) of more than 40 data-poor PFAS and then ranked likelihood of bioaccumulation. Emerging PFAS such as perfluoroalkylamides and fluorotelomer halides were observed to have less affinity for plasma proteins, and in turn have lower bioaccumulation potential, compared to higher binding compounds such as polyfluorinated amines and perfluorohalides [12]. Ryu et al. [13] took the opposite and novel approach of evaluating which PFAS show the least binding and are unbound in the serum. This study showed that rats and humans have very similar unbound fractions across the different PFAS tested, with the fraction in mice slightly higher. Molecular weight (MW) was observed to be inversely associated with being unbound (i.e., greater MW = more bound), with PFHxS having a smaller unbound fraction than expected. The PFAS with the greatest unbound fraction of those tested were the 4-carbon PFAS, PFBA and PFBS, which have almost 10% unbound fractions and in turn much shorter half-lives, as expected based on MW [13].

Transporters are also important in the bioaccumulation of some PFAS. There are few models, either in vivo or in vitro, that demonstrate differences in bioaccumulation. Williams et al. [14] used one of the models to correlate PFOS bioaccumulation, with increased *oatp1a4-6* members in mouse liver and to a lesser extent *Fatp1*, *asbt*, and *slc22a26*. The roles of *fatp1* and *slc22a26*, an ortholog of SLC22A25 in humans, have not been evaluated as PFAS transporters.

Due to the predicted long half-lives and bioaccumulation of certain PFAS, toxicokinetics (TK) research is increasing. The prime subjects of studies have been PFOA and/or PFOS. Toxicokinetics of PFHxS and PFNA have also been studied more than emerging PFAS. East et al. [11] assessed the current literature on applications of TK modeling for PFAS and noted that most are one-compartment models of human exposure to PFOA and PFOS. Moving to more complex models, such as physiologically-based toxicokinetic (PBTK) models with legacy PFAS, new short-chain PFAS, or PFAS alternatives, could be an informative new step over the next 10 years [11].

PFAS are rarely found as a single entity and instead are primarily produced and released as mixtures [16]. Three studies in the Special Issue evaluated PFAS mixture toxicity [17,18,19]. A mixture of commonly occuring PFAS with relevance to human exposure was used at environmentally relevant concentrations to evaluate toxicity after 5 weeks of exposure in mice. Liver vacuolization and hypertrophy were associated with changes in gene expression related to energy metabolism and cancer; phospholipid metabolism and glutathione concentrations were also perturbed. Several changes in gene expression are consistent with PPAR activation; however, other nuclear receptor pathways were also perturbed [18]. In addition, exposure of pregnant females to a mixture of legacy PFAS caused significant liver damage in offspring and proteomic changes associated with drug-drug interactions, lipid fatty acid transport storage, oxidation, and synthesis. These data indicate that adverse effects associated with metabolic disease may occur due to PFAS exposure from the mother during pregnancy or lactation [19]. PFAS mixture effects were also observed in a marine model, the sheepshead minnow (*C. variegatus*). Toxicity was associated with PPAR activation and oxidative stress. Interestingly, PFOA antagonized PFOS toxicity by reducing PFOS-mediated oxidative stress [17]. This finding supports other in vitro work [20] that demonstrates PFOA antagonized PFOS toxicity in vitro but contrasts with findings by Conley et al. [21] that indicated in vivo co-exposure to PFOA and PFOS produced cumulative effects.

Several studies in the Special Issue demonstrated hepatic, immune/inflammation, and endocrine toxicity, much of which included significant effects on lipid metabolism [14,22,23,24]. One study investigated metabolic and microbiome changes. Within the metabolome, lipid synthesis, steroid synthesis, bile acid metabolism, and nitrogen and amino acid metabolism were perturbed in a manner consistent with hepatoxicity and metabolic disorders. The two PFAS studied, HFPO-DA (commonly referred to as “GenX”) and PFOS, had very similar effects on metabolic pathways. However, PFOS had a much greater effect on *Lactobacillus* and *Limosilactobacillus* in addition to other metabolite and microbiome alterations [22].

Two other rodent studies investigated two different sub-classes of PFAS—a perfluorinated carboxylic acid, PFOS [14], and a perfluoroether carboxylic acid, HFPO-TeA, a replacement PFAS [23], and observed problematic weight loss or wasting associated with the PFAS treatment. In addition, the females were more sensitive to the toxic effects of the PFAS. HFPO-TeA induced liver hypertrophy in association with depressed thyroid hormones [23]. PFOS induced liver hypertrophy and steatosis in association with increased inflammatory markers such as PGE2 as well as changes in oxylipin di-hydroxy to epoxide ratios. Other observations included significant perturbations in lipid metabolism and transport and storage genes in the liver, including *fabp1*, which encodes a fatty acid binding protein that retains PFAS [14]. Overall, this study provides potential lipidomic biomarkers for PFOS toxicity and genes that play a role in interindividual differences in bioaccumulation in mice.

A comparative and crucial transcriptomic study of more than 2000 samples evaluated whether there were conserved transcriptomic profiles across diverse species and different PFAS [24]. It found and confirmed for the first time that hormone response, immune response, and lipid metabolism pathways are commonly perturbed by diverse sub-classes of PFAS and across different species, such as humans, mice, nematodes, and fish [24]. This suggests the mode of action of many PFAS are similar, and that environmental and human relevant toxicology models may be key tools to be used in hazard or risk assessments for integrative toxicology approaches such as the One Health initiative by the World Health Organization (WHO) [25].

Models other than human and rodent also were tested, and the results are reported in this Special Issue. These included *C. variegatus* (sheepshead minnow) [17], *D. rerio* (zebrafish) [26,27], and *C. elegans* (nematode) [28] and other aquatic species [29]. Studies with the nematode, *C. elegans*, observed that PFAS are obesogenic, neurotoxic, immunotoxic, and cause developmental delays [28], all of which are commonly evaluated toxic endpoints in multiple studies with fish, mice, and human in vitro models and epidemiology studies. Using zebrafish as a developmental and behavioral toxicology model showed increases in developmental deformities and embryo activity and decreases in lipid levels and visual responses upon exposure to PFOS, PFOA, and PFHxS. The magnitude of the response was greatest for PFOS and least for PFOA [27]. Britton et al. [26] used zebrafish as a medium throughput model for developmental toxicity of 183 PFAS. Thirty PFAS caused significant developmental toxicity, although there was no discernable pattern based on PFAS structure. Interestingly, while PFOS was one of these toxic chemicals, PFOS precursors such as PFOSA and N-MeFOSA were significantly more toxic. Few of these chemicals have been tested in mammalian studies; further studies on PFOSA and N-MeFOSA in particular may be warranted [26]. Lastly, Chung et al. [29] evaluated the effects of temperature and salinity on the toxicity of PFOS in multiple marine or estuarine species, such as *C. variegatus* (sheepshead minnow), *T. obsoleta* (Eastern mud snails), *P. pugio* (grass shrimp), and *A. bahia* (mysids). Higher temperatures greatly increased toxicity in the sheepshead minnow, with other species showing less sensitivity. Higher salinity greatly decreased toxicity in the Eastern mud snail, but less so in other species. This work demonstrates that changing environmental factors are likely to affect PFAS toxicity with differences across species [29]. These environmental effects may need to be considered within estuarine ecosystems.

## 3. Conclusions

**Figure 1:**
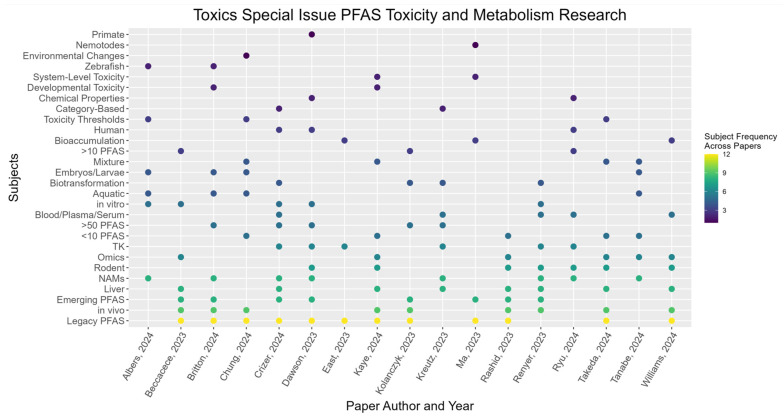
Selected topics from the Special Issue PFAS Toxicology and Metabolism.

As described above, the subjects of the 17 papers published in this Special Issue present cutting-edge research in several different areas that enhance our understanding of toxicity and metabolism of PFAS. (See Figure 1) The enormous number of PFAS, the known potential for toxicity, and the lack of data for many PFAS suggest data gaps continue to exist. Our understanding of the health and environmental effects of PFAS exposure would likely benefit from future research in the following areas: (1.) development and use of methods for rapid toxicity assessment, including for emerging PFAS which have little available toxicity data; (2.) design of exposure studies to reflect PFAS occurrence in mixtures; (3.) use of analytical approaches for gathering biological systems-level exposure; (4.) development of targeted and non-targeted analytical methods to identify and quantitate a greater number of PFAS, especially to identify those occurring in humans and the environment; (5.) cross-species studies; (6.) analysis to evaluate the presence of precursor PFAS and biotransformations; and (7.) creation of tools for organizing and understanding data. New approach methods (NAMs) can quickly evaluate toxicity for large numbers of PFAS or gather high throughput data to enable more timely risk assessment. Examples include in vitro toxicokinetics studies for large numbers of PFAS [12,15] and across species [13], in vivo studies coupled with high throughput TK and/or omics methods [14,17,18,19,22,24], and toxicity evaluations that utilize fish or invertebrate embryos and larvae as models [17,26,27,29]. These assays can generate data on biological interactions of multiple PFAS in less time than is typically needed for traditional in vivo exposure studies.

PFAS are often detected as mixtures in water, soils, foods, food packaging, clothing, and a variety of consumer products, leading to human exposure via multiple pathways [30,31]. This distribution highlights the need to characterize combinations of PFAS together [17,18,19,29]. Many NAMs currently focus on individual chemicals for each assay, but assays to test mixtures could yield greater understanding of real-world exposures. NAMs assays with human and other species cell lines may produce comparative results that are important for extrapolation of impacts and provide further support for reductions in the use of animal models [23]. Exposomics studies using non-targeted analysis (NTA) [32], which can generate qualitative data for potentially thousands of chemicals present in a sample, could be especially beneficial for characterizing PFAS in biomonitoring and identification of precursors and biotransformation products. Future studies could use NTA to explore exposure to mixtures of PFAS from environmental sources. The number of PFAS selected by targeted mass spectrometry methods could be expanded for accurate quantitation of emerging compounds, though low sample volumes and availability of commercial standards present significant barriers. The lack of authentic standards can be largely overcome with NTA, however quantitation of PFAS at the low concentrations detected in humans is currently a challenge.

PFAS are known to affect multiple biological systems and pathways [30]. Further understanding of the systems-level impacts of PFAS exposure could be enhanced by greater use of omics (transcriptomics, proteomics, metabolomics, lipidomics) and research that combines and integrates multiple omics approaches for both legacy and emerging PFAS. Four papers in this issue demonstrate the success of omics approaches for clarifying biological impacts of exposures to legacy PFAS [18,19,22,33]. Omics studies involving exposures to mixtures and/or novel, emerging PFAS could help address current concerns that replacement PFAS may be as toxic as the original formulations. PFAS are commonly known as “forever” chemicals and researchers are finding precursors that transform through abiotic and/or biotic pathways to non-reactive, bioaccumulative species such as PFOS and PFOA [12,15,34]. Research into the occurrence of precursors in the environment and biological organisms could enable prioritization of chemicals for risk assessment. Databases [10] for organizing and models for extrapolating [9,11] the burgeoning volume of published PFAS data could also provide decision-makers with tools necessary to protect human health and the environment.
